# Clonal analysis of fetal hematopoietic stem/progenitor cells reveals how post-transplantation capabilities are distributed

**DOI:** 10.1016/j.stemcr.2024.07.003

**Published:** 2024-08-01

**Authors:** Olivia J. Stonehouse, Christine Biben, Tom S. Weber, Alexandra Garnham, Katie A. Fennell, Alison Farley, Antoine F. Terreaux, Warren S. Alexander, Mark A. Dawson, Shalin H. Naik, Samir Taoudi

**Affiliations:** 1 The Walter and Eliza Hall Institute of Medical Research, Melbourne, Victoria, Australia; 2 The University of Melbourne, Melbourne, Victoria, Australia; 3 School of Cellular and Molecular Medicine, University of Bristol, Bristol, England, UK; 4 Sir Peter MacCallum Department of Oncology, The University of Melbourne, Melbourne, Victoria, Australia; 5 The University of Melbourne Centre for Cancer Research, The University of Melbourne, Melbourne, Victoria, Australia; 6 Lowy Cancer Research Centre, UNSW, Sydney, New South Wales, Australia

## Abstract

It has been proposed that adult hematopoiesis is sustained by multipotent progenitors (MPPs) specified during embryogenesis. Adult-like hematopoietic stem cell (HSC) and MPP immunophenotypes are present in the fetus, but knowledge of their functional capacity is incomplete. We found that fetal MPP populations were functionally similar to adult cells, albeit with some differences in lymphoid output. Clonal assessment revealed that lineage biases arose from differences in patterns of single-/bi-lineage differentiation. Long-term (LT)- and short-term (ST)-HSC populations were distinguished from MPPs according to capacity for clonal multilineage differentiation. We discovered that a large cohort of long-term repopulating units (LT-RUs) resides within the ST-HSC population; a significant portion of these were labeled using *Flt3*-cre. This finding has two implications: (1) use of the CD150+ LT-HSC immunophenotype alone will significantly underestimate the size and diversity of the LT-RU pool and (2) LT-RUs in the ST-HSC population have the attributes required to persist into adulthood.

## Highlights

• Functional equivalents of adult stem/progenitor cells exist in the fetal liver
• Large numbers of *bona fide* LT-RUs reside in the fetal ST-HSC population
• *Flt3*-cre tracking indicated that multiple LT-RU-forming pathways exist

## Teaser

In this article, Taoudi and colleagues show that a large contingent of long-term repopulating cells covertly resides within an immunophenotype previously thought to contain lower-end progenitor cells. This indicates that over-reliance on the conventional stem cell immunophenotype will underestimate the ontological and functional diversity of the stem cell pool.

## Introduction

Hematopoiesis ensures the continuous supply of mature blood. Whether the adult hematopoietic hierarchy is stem cell driven or is a process sustained by multipotent progenitor cells (MPPs) but underwritten by hematopoietic stem cells (HSCs) is debated (Busch et al., 2015; Pei et al., 2017, 2020; Rodriguez-Fraticelli et al., 2018; Sawai et al., 2016; Sawen et al., 2018; Sun et al., 2014). Based on the ability to provide long-term multilineage reconstitution following transplantation, HSCs are traditionally thought to be at the foundation of the hierarchy. Adult bone marrow (ABM) HSCs consist of subsets classified according to durability of self-renewal and by lineage production (Dykstra et al., 2007; Eaves, 2015; Muller-Sieburg et al., 2002; Oguro et al., 2013; Pietras et al., 2015). HSCs and MPPs are most commonly distinguished according to variations in expression of lineage marker (LIN)− SCA1+ KIT+ (LSK) and SLAM systems, known as the LSK-SLAM code (Kiel et al., 2005; Pietras et al., 2015). Long-term reconstituting HSCs (LT-HSCs) are recognized as LSK_FLT3−CD150+CD48− cells, and short-term reconstituting HSCs (ST-HSCs) are LSK_FLT3−CD150−CD48− (Kiel et al., 2005; Pietras et al., 2015).

MPPs are downstream of HSCs. ABM MPPs are capable of acute-term multilineage differentiation when transplanted into irradiated adult mice (Adolfsson et al., 2001; Kiel et al., 2005; Oguro et al., 2013; Pietras et al., 2015). There are three commonly studied MPPs subclasses: MPP2, MPP3, and MPP4. Each ABM MPP subset is capable of lymphoid, myelo-erythroid, and megakaryocyte/platelet production but exhibits lineage biases (Morales-Hernandez et al., 2018; Pietras et al., 2015): MPP2 (LSK_FLT3−CD150+CD48+) and MPP3 (LSK_FLT3−CD150−CD48+) exhibit limited lymphoid fate; MPP2 exhibits erythroid and megakaryocyte/platelet bias; MPP3 exhibits granulocytic bias; and MPP4 (LSK_FLT3+CD150−CD48+) exhibits a lymphoid bias.

*In situ* barcoding studies suggest that long-term self-renewing MPPs are the drivers of native hematopoiesis because the barcodes present in mature lineages are found in the MPPs but not in LT-HSCs (Pei et al., 2017; Sun et al., 2014).

Understanding the biology of HSCs and MPPs during embryogenesis is important because this is when the lineages emerge (Medvinsky et al., 2011; Patel et al., 2022). The first long-term repopulating units (LT-RUs, a functional designation rather than an immunophenotypic description) express KIT (Sanchez et al., 1996) and SCA1 (de Bruijn et al., 2002), but they lack CD150 expression (McKinney-Freeman et al., 2009). By E14.5, LT-RUs in the fetal liver (FL) are CD150+ (Kim et al., 2006). Cells with the ST-HSC immunophenotype (CD150−) are present in the E14.5 FL (Kim et al., 2006; Patel et al., 2022), but their functional capacity has not been thoroughly investigated. Although it is known that long-term hematopoietic reconstitution is possible from CD150− cells in the E14.5 FL (Kent et al., 2009; Kim et al., 2006; Papathanasiou et al., 2009), how much of the LT-RU biomass they contain is not known. This is an important knowledge gap because of how ubiquitously CD150 expression is used as a hurdle criterion for the investigation of fetal LT-RUs.

It has been proposed that the first phase of HSC-derived blood production could be contributed to by developmentally restricted HSCs (drHSCs) (Beaudin et al., 2016). drHSCs derive along an *Flt3-Cre*-expressing ancestry, express the conventional LT-HSC immunophenotype (are CD150+), and provide durable but lymphoid-biased reconstitution. drHSCs differ from LT-HSCs according to their ancestry and their longevity under physiological conditions (Beaudin et al., 2016). One interpretation is that drHSCs represent the prenatal equivalent of ST-HSCs (Patel et al., 2022).

How functionally comparable FL and ABM MPPs are remains incompletely understood. Cells with an MPP2 immunophenotype from the E16.5 and E18.5 FL showed evidence of transient multilineage reconstitution (Hall et al., 2022). MPP4-like features such as lymphoid transcriptional priming and lymphoid differentiation bias have been reported in the E14.5 FL (Benz et al., 2012; Kim et al., 2006; Mansson et al., 2007).

We used single-cell RNA sequencing (scRNA-seq), cellular barcoding, and transplantation experiments to investigate E14.5 FL HSCs and MPPs. We found that functionally diverse MPP subtypes do exist within the embryo. Tracking post-transplantation fate using clonal technology revealed that multilineage outcomes of MPPs were achieved by combinations of single- or dual-lineage contribution. Most intriguingly, we discovered that two variants of *bona fide* LT-RUs (without overt lineage bias) co-exist in the FL, one is within the LT-HSC population and the other within the ST-HSC population. Strikingly, the majority of LT-RUs reside in the ST-HSC population. Using *Flt3*-cre lineage tracking we found that ST-HSCs were more significantly labeled than LT-HSCs. This suggests that, in addition to the existence of ontologically independent pathways of LT-RU and progenitor cell emergence (Yokomizo et al., 2022), multiple LT-RU-forming pathways exist. In combination with the insight that embryonic *Flt3*-cre-labeled cells contribute to native adult hematopoiesis (Patel et al., 2022), our findings suggest that LT-RUs present in the FL ST-HSC population could persist into adulthood and contribute to native hematopoiesis.

## Results

### Quantitative and transcriptional investigation of fetal LSK subsets

**Figure 1:**
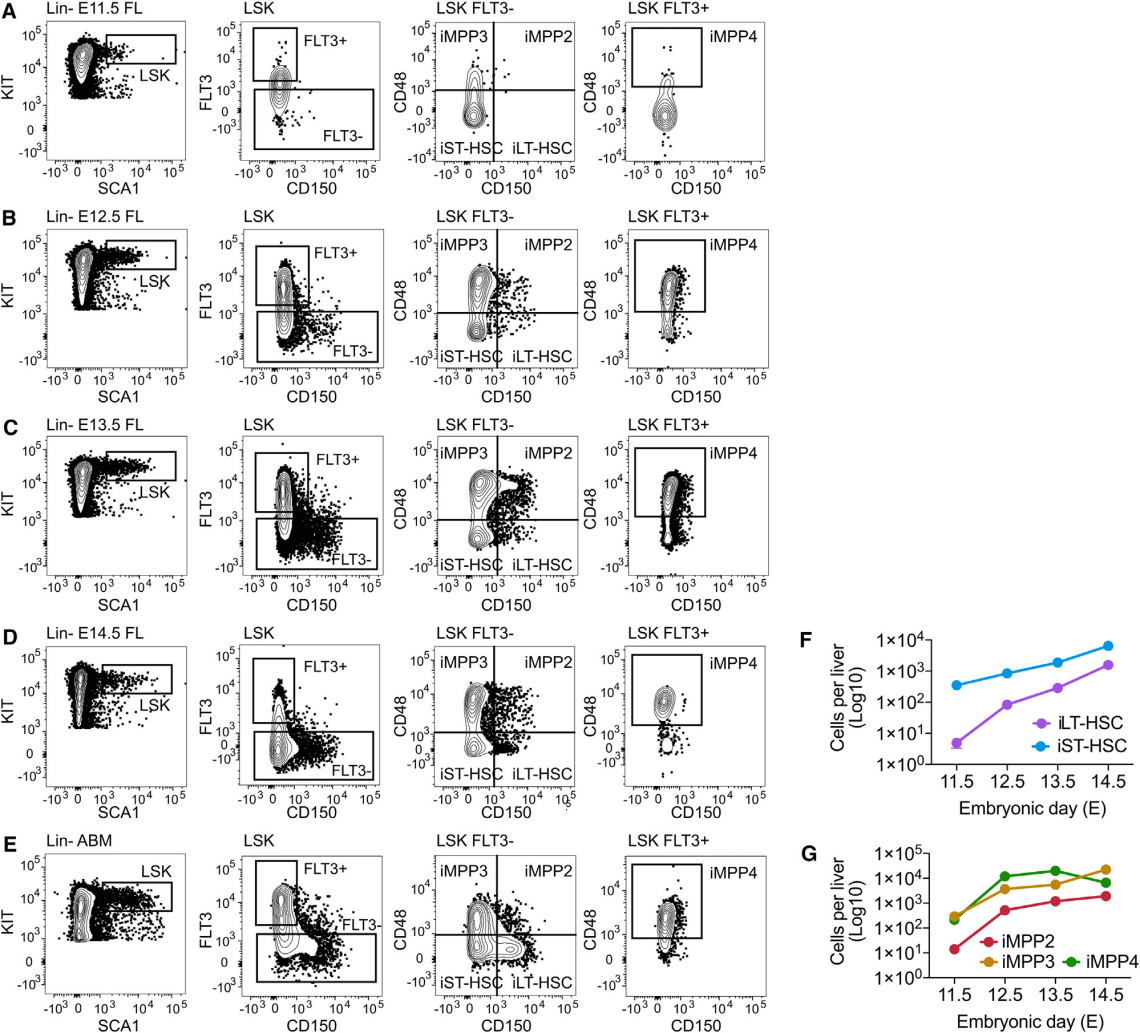
Quantification of LSK subsets (A–E) Representative plots of LSK subsets in E11.5 (A), E12.5 (B), E13.5 (C), and E14.5 (D) fetal liver (FL), and the adult bone marrow (E). (F) Quantification of E14.5 iLT-HSC and iST-HSC populations. E11.5 *n* = 7 embryos; E12.5, E13.5, and E14.5 *n* = 6 embryos per stage. Values, mean ± SD. (G) Quantification of E11.5–E14.5 FL iMPPs. E11.5 *n* = 7 embryos; E12.5, E13.5, and E14.5 *n* = 6 embryos per stage. Values, mean ± SD. Samples for each developmental stage were collected from ≥3 litters.

Adult LSK-SLAM immunophenotypes (Pietras et al., 2015) were used to investigate the appearance of HSC/MPP subsets in the E11.5–E14.5 FL (Figures 1A–1D). To avoid the presumption of functional equivalence to ABM cells, we adopted the convention of referring to cell populations with the immunophenotypic (“i”) prefix (e.g., iST-HSC), as has been implemented previously by others (Chen et al., 2016; Dong et al., 2020). At E11.5, iST-HSC, iMPP3, and iMPP4 populations were observed (Figures 1A–1G). By E12.5, all LSK subtypes, including iLT-HSCs and iMPP2, were detected (Figures 1B–1G).

**Figure 2:**
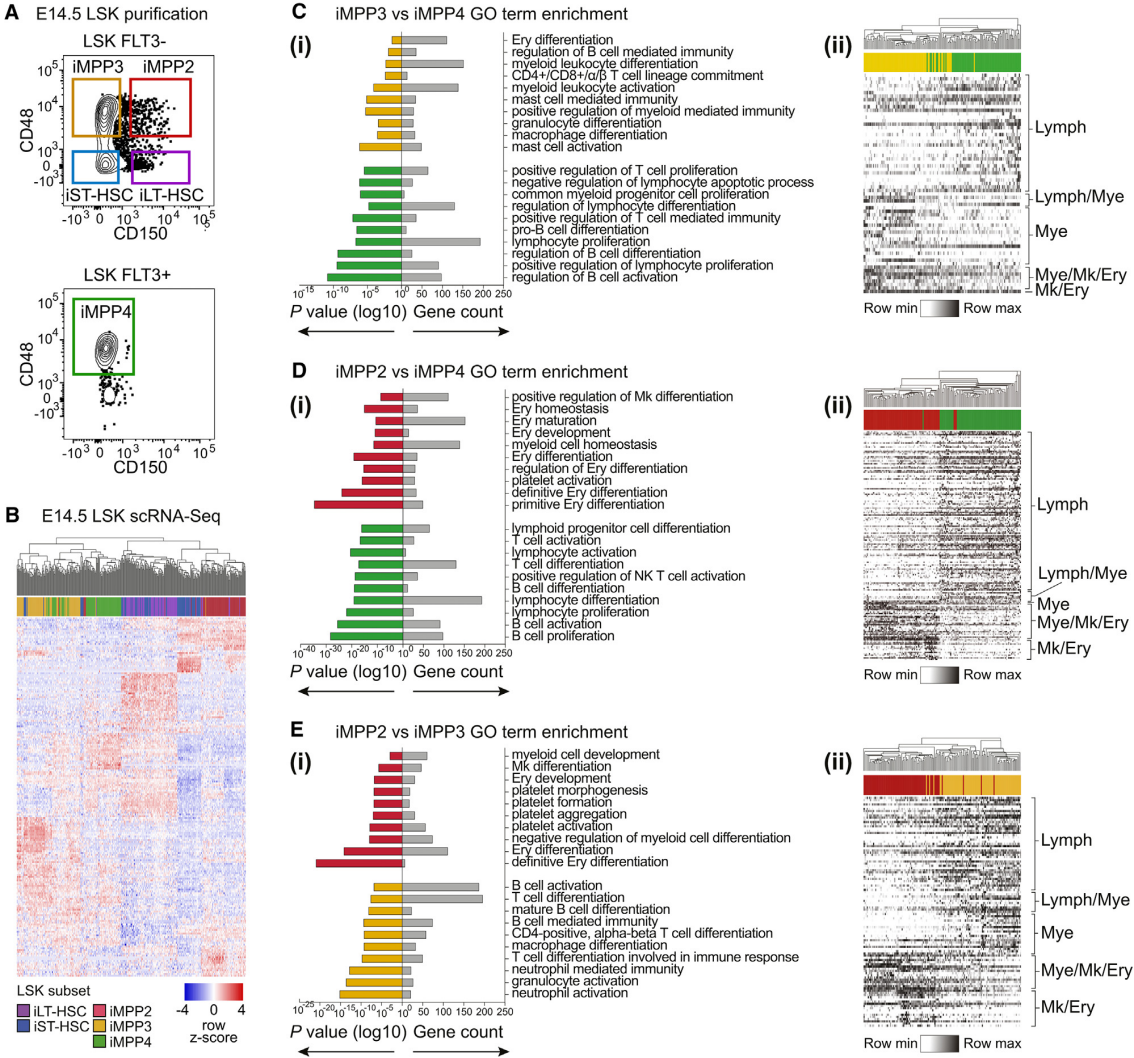
scRNA-seq of E14.5 iHSCs and iMPPs (A) Gating strategy used to purify E14.5 FL LSK subsets. (B) Heatmap of 100 most variable genes for each LSK subtype. (C) Gene Ontology (GO) term enrichment analysis for differentially expressed genes for iMPP3 versus iMPP4 (i). (ii) Genes in significant GO terms. (D) GO term analysis for iMPP2 versus iMPP4 (i). (ii) Genes in significant GO terms. (E) GO term analysis for iMPP2 versus iMPP3 (i). (ii) Genes in significant GO terms. Lymph, lymphoid; Lymph/Mye, lymphoid and myeloid; Mye, myeloid; Mk/Ery, megakaryocyte and erythroid; Mye/Mk/Ery, myeloid, megakaryocyte and erythroid.

We compared the transcriptomes of E14.5 FL LSK subsets to gain insight into potential functional differences. To this end, single cells were purified (Figure 2A) using indexed flow cytometry (which recorded the immunophenotype of the purified cell). Index data were used to verify the immunophenotype of the purified cells. Unsupervised hierarchical clustering of scRNA-seq data indicated that E14.5 iMPP2–4 were transcriptionally distinct subsets (Figure 2B). Gene Ontology (GO) term analysis using significantly differentially expressed genes between iMPP2–4 revealed the following.

• Enrichment of pathways related to lymphoid differentiation/function in iMPP4 (Figures 2Ci and 2Di; Tables S1 and S2). This was in keeping with the lymphoid bias of ABM iMPP4 (Pietras et al., 2015).
• Enrichment of pathways associated with megakaryocytes and erythroid differentiation in iMPP2 (Figures 2Di and 2Ei; Tables S2 and S3). This was consistent with the megakaryocyte/erythroid bias of ABM iMPP2.
• Relative to iMPP2 and iMPP4, iMPP3 exhibited a more lineage-balanced transcriptional profile which included features of erythro-myeloid, megakaryocytic, and lymphoid lineages (Figures 2Ci and 2Ei; Tables S1 and S3).

Visualization of genes associated with enriched GO terms confirmed that they were broadly expressed within the relevant populations (Figures 2Cii–Eii, Table S4). Thus, adult MPP immunophenotypes identify fetal cells with transcriptional signs of lineage priming consistent with the bias of their ABM immunophenotypic counterparts.

### Post-transplantation comparison of E14.5 and ABM immunophenotypic counterparts

To compare the acute-term *in vivo* differentiation potential of E14.5 FL and ABM LSK subtypes, cells purified from UBC-GFP mice were transplanted into GFP− sub-lethally irradiated recipients. To enable a like-for-like comparison, the number of cells transplanted was determined by the availability of cells in the E14.5 FL, and the number of cells that would yield robust engraftment. From either E14.5 FL or ABM donors, 1,200 iLT-HSCs, 4,000 iST-HSCs, 7,000 iMPP2s, 25,000 iMPP3s, and 30,000 iMPP4s were transplanted. *In vivo* fate was assessed after two weeks. This time point was selected because it allows the identification of lineage biases if they exist (Morales-Hernandez et al., 2018; Pietras et al., 2015) and ensures that the peak output of iMPPs (which rapidly exhaust [Morales-Hernandez et al., 2018; Pietras et al., 2015]) would be captured. Contribution to platelets, erythrocytes, myeloid, and lymphoid lineages was assessed in the recipient peripheral blood, spleen, and thymus (Figure S1).

**Figure 3:**
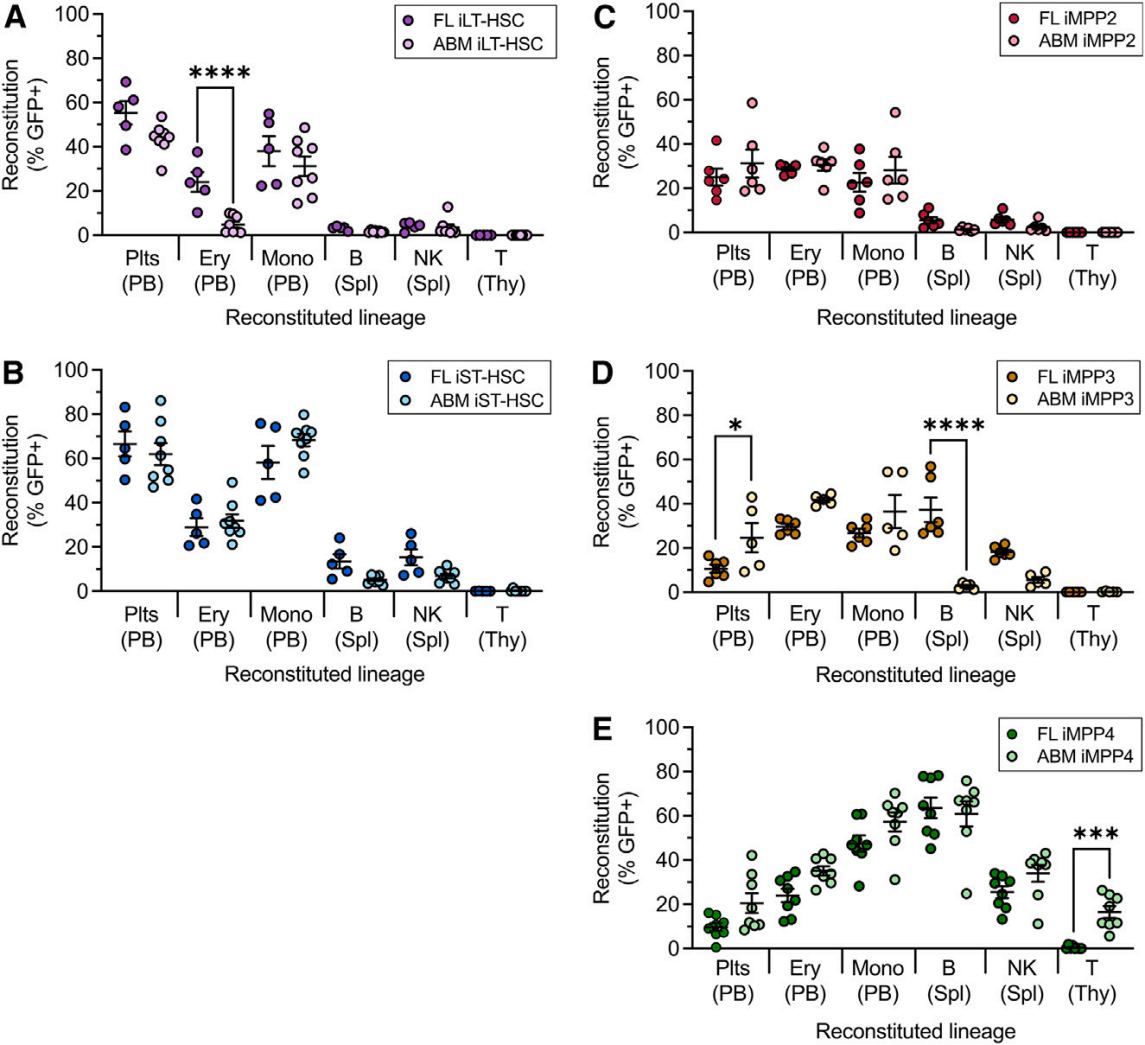
Functional comparison of E14.5 LSK subsets with ABM counterparts Comparison of reconstitution of sub-lethally irradiated recipients by E14.5 and ABM iLT-HSCs (A), iST-HSCs (B), iMPP2 (C), iMPP3 (D), and iMPP4 (E) 2 weeks after transplantation. Only values *p* < 0.05 are shown. ^∗^, *p* < 0.05. ^∗∗^, *p* < 0.005, ^∗∗∗^, *p* < 0.0005, ^∗∗∗∗^, *p* < 0.00005. ABM iLT-HSC: *n* = 8 recipients, 3 donors, 3 experimental days. ABM iST-HSC: *n* = 8 recipients, 3 donors, 3 experimental days. E14.5 iLT-HSC: *n* = 5 recipients, donor cells from pools of livers from 2 litters, 2 experimental days. E14.5 iST-HSC: *n* = 5 recipients, donor cells pools from livers from 2 litters, 2 experimental days. ABM iMPP2: *n* = 6 recipients, 4 donors, 4 experimental days. ABM iMPP3: *n* = 5 recipients, 4 donors, 4 experimental days. ABM iMPP4: *n* = 8 recipients, 4 donors, 4 experimental days. E14.5 iMPP2: *n* = 6 recipients, donor cells pooled from livers from 4 litters, 4 experimental days. E14.5 iMPP3: *n* = 6 recipients, donor cells collected from pools of livers collected from 4 litters, 4 experimental days. E14.5 iMPP4: *n* = 8 recipient mice, donor cells from pools of livers from 4 litters, 4 experimental days. Plt, platelet; Ery, erythroid; Mono, monocyte; B, B cell; T, T cell; NK, natural killer cell; Parentheses, analyzed tissue; PB, peripheral blood; Spl, spleen; Thy, thymus. For (A)–(E), donor-derived reconstitution (Reconstitution %) was determined using donor-specific GFP expression.

Inter-developmental stage comparison of the HSC subsets revealed similar outcomes (Figures 3A and 3B); the exception was greater erythroid output by FL iLT-HSCs (Figure 3A). Of note, the low reconstitution of T cells observed after 2 weeks was consistent with previous studies (Forsberg et al., 2006; Papathanasiou et al., 2009; Pietras et al., 2015). iMPP subsets also performed similarly between the developmental stages (Figures 3C–3E). The exceptions were enhanced B cell output from FL iMPP3 (Figure 3D) and diminished T cell output from FL iMPP4 (Figure 3E). We note that failure to produce a lineage at the 2 week time point does not necessarily indicate a lack of potential.

### Clonal tracking post-transplantation fates of E14.5 iHSC and iMPP subsets

To understand the clonal structure of post-transplantation reconstitution, we transplanted iHSC and iMPP subsets following lentiviral cellular barcoding. *Ex vivo* cellular barcoding involves the indelible labeling of individual cells with a high-diversity library of genetically heritable DNA sequences termed barcodes (Schepers et al., 2008). Once transduced, barcode-carrying cells are transplanted into irradiated recipients and their barcoded progeny can be purified by flow cytometry. Clonal ancestry can be determined by retrieving barcode sequences from the genome of donor-derived cells.

**Figure 4:**
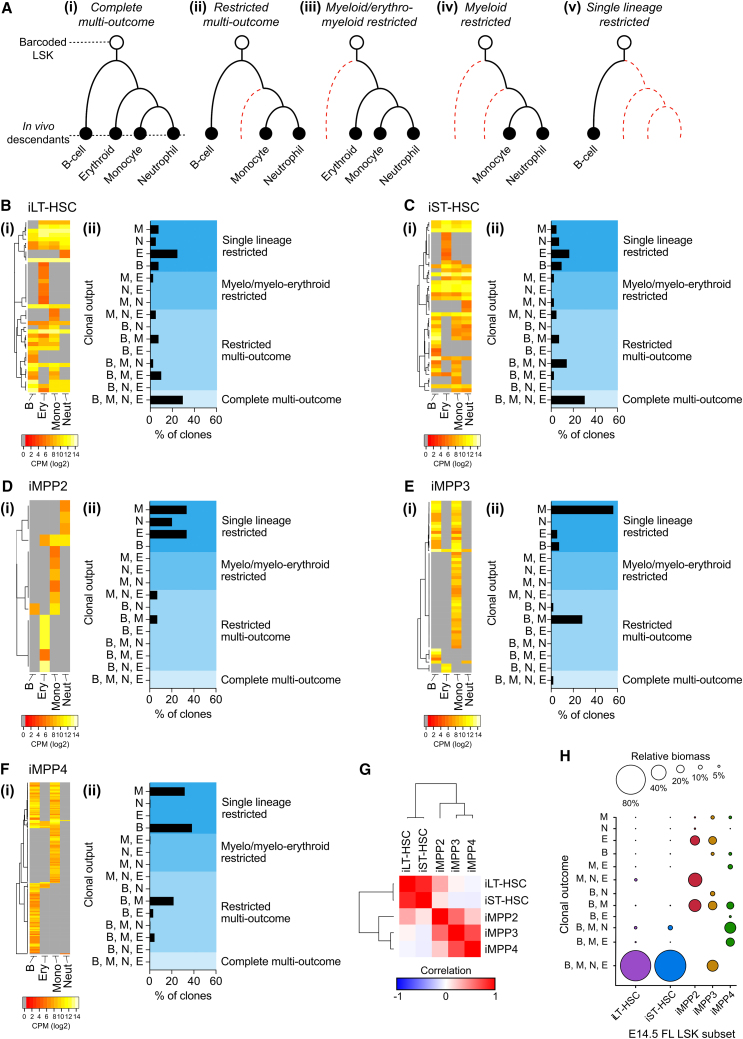
Clonal tracking post-transplantation E14.5 LSK fates (A) Clonal fate classifications after 2 weeks: (i) Complete multi-outcome, a barcode contributes to all lineages; (ii) Restricted multi-outcome, a barcode contributes to B cells and at least one erythro-myeloid lineage; (iii) Erythro-myeloid restricted, a barcode in erythroblasts, monocytes, and granulocytes; (iv) Myeloid restricted, a barcode in monocytes and granulocytes; (v) Single-lineage restricted, barcode contribution to only one lineage. (B–F) Heatmaps of barcode reads (i) and histograms summarizing the frequencies of clonal fates (ii) for iLT-HSC (B, *n* = 41 clones), iST-HSC (C, *n* = 43 clones), iMPP2 (D, *n* = 15 clones), iMPP3 (E, *n* = 57 clones), and iMPP4 (F, *n* = 168 clones). (G) Similarity matrix analysis of clonal outcomes. (H) Bubble plots of the proportion of reads associated with specific clonal outcomes (relative biomass). iLT-HSC: *n* = 4 recipients, donor cells from pools of 4 litters, 4 experimental days. iST-HSC: *n* = 3 recipients, donor cells pooled from 2 litters, 2 experimental days. iMPP2: *n* = 2 recipient, donor cells pooled from two litters, 2 experimental days. iMPP3: *n* = 2 recipients, donor cells pooled from 2 litters, 2 experimental days. iMPP4: *n* = 3 recipients, donor cells pooled from 3 litters, 3 experimental days. M, monocyte; N, neutrophil; E, erythroid cell; B, B cell; CPM, counts per million reads.

Purified E14.5 subsets were transduced with a previously described mCHERRY-expressing lentiviral barcode library composed of ∼7 × 10^4^ unique barcodes (Fennell et al., 2022). A maximum of 3 × 10^4^ cells were cultured with a pre-titered library for 16 h to achieve 1%–2% transduction. This provided a >100-fold excess in barcode diversity, making repeat use of barcodes and multiple infection of individual cells unlikely (Kebschull and Zador, 2018; Naik et al., 2014) (Figure S2). Of note, the culture period did not affect *in vivo* output (Figure S2). Two weeks after transplantation, mCHERRY+ B cells, erythroblasts, monocytes, and granulocytes were collected from recipients. At 2 weeks post-transplantation, the spleen not only contains the offspring of clones that also engraft the ABM but also contains clones that have not engrafted the ABM (Naik et al., 2013). Accordingly, to ensure effective retrieval of barcoded offspring, we collected lineages from the spleen (Figure 4A).

E14.5 iLT- and iST-HSCs underwent similar clonal fates (Figures 4B and 4C). This ranged from complete multilineage outcomes that spanned the lymphoid, myeloid, and erythroid lineages (∼30% of clones); various combinations of restricted multi-outcomes; erythro-myeloid restriction; and single-lineage restriction (Figures 4B and 4C). In stark contrast, clonal tracking of iMPPs revealed the following.

• Of iMPP2 clones, some were capable of restricted multi-outcomes, but 86% of clones underwent single-lineage myeloid or erythroid production (Figure 4D).
• Of iMPP3 clones, 68% were single-lineage restricted; this included production of B cells, monocytes, or erythroblasts. 30% of clones underwent restricted multi-outcomes (myeloid lineage and B cell outcome), and only 1 clone underwent complete multi-outcome (Figure 4E).
• iMPP4 clones were more prone to B cell-restricted outcome than iMPP3 (Figure 4F).
• iMPPs subsets were all capable of erythropoiesis but differed according to how this was accomplished. iMPP2-derived erythropoiesis occurred from erythroid-only and erythroid-macrophage-restricted differentiation (Figures 4D and 4H). iMPP3-derived erythropoiesis occurred from erythroid-only and complete multi-outcome clones (Figures 4E and 4H). iMPP4-derived erythropoiesis occurred from lymphoid-erythroid-restricted and restricted multi-outcome clones (Figures 4F and 4H).
• Although all iMPPs exhibited some degree of multilineage outcomes, contribution to all tested lineages was a feature largely restricted to the HSCs.

Correlation of outcomes between LSK subsets revealed a marked difference between iHSCs and iMPPs (Figure 4G). This distinction was also evident when contribution of clones to lineage biomass was considered (Figure 4H).

These data revealed that E14.5 iLT- and iST-HSCs contained cells with the capacity for complete multilineage outcomes and that the lineage-biased outcomes observed from MPPs at the population level were likely driven by a mix of clones that underwent with single-lineage-restricted outcomes.

### Comparison of stemness between E14.5 FL iLT- and iST-HSCs

From the barcoding experiments, we noted that iLT- and iST-HSCs performed remarkably similarly. ABM iST-HSCs diverge from ABM iLT-HSCs according to durability of multilineage reconstitution and by the inability to self-renew (Morita et al., 2010; Oguro et al., 2013; Pietras et al., 2015). To better understand E14.5 iST-HSCs, we next compared them to E14.5 iLT-HSCs in long-term transplantation assays. To distinguish immunophenotypic classification from stem cell function, we will continue to refer to immunophenotypes as either iLT-HSC or iST-HSC, but the provision of durable multilineage reconstitution will be referred to as the output of a long-term repopulating unit (LT-RU).

**Figure 5:**
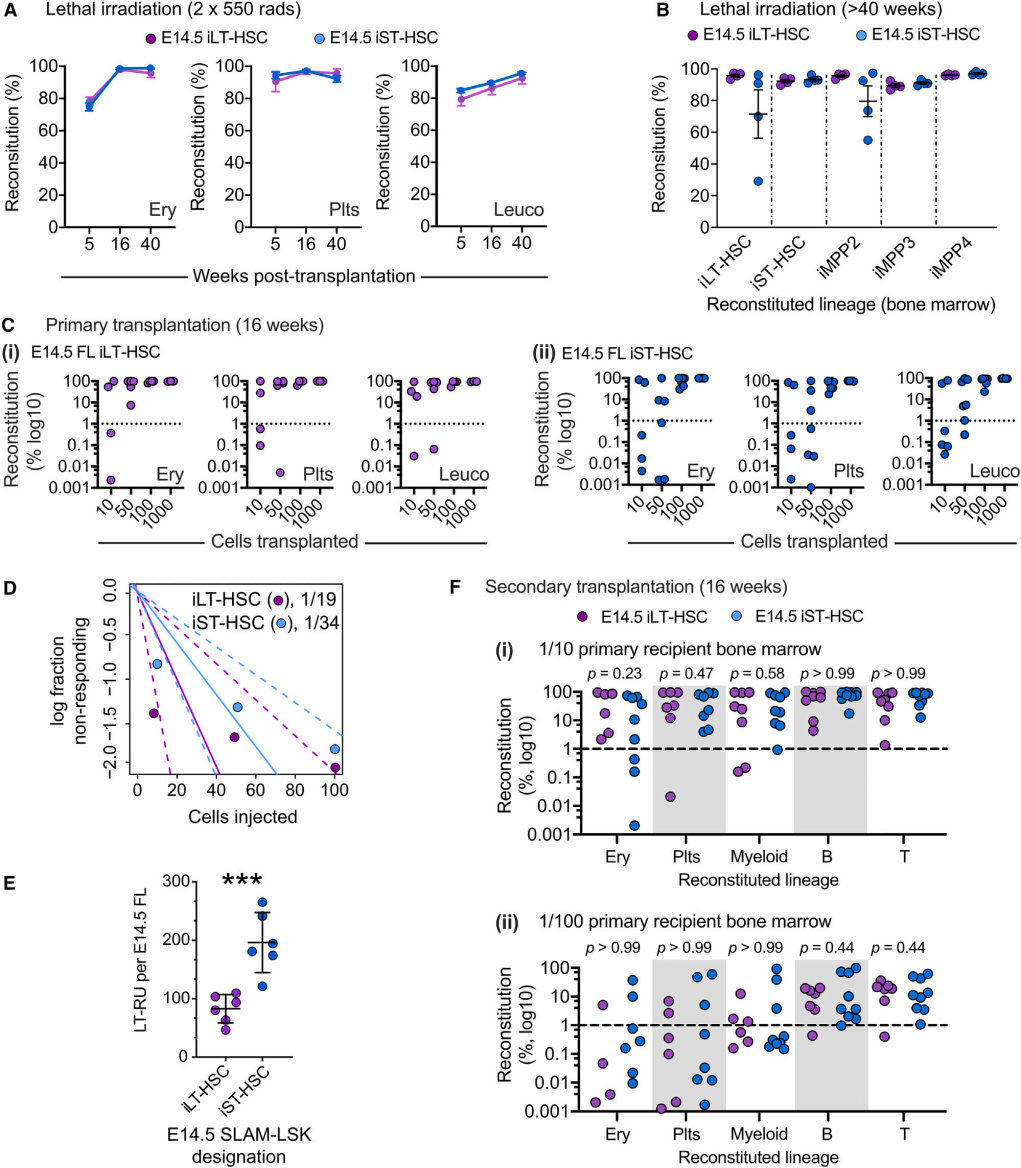
Discovery of large numbers of LT-RUs in the iST-HSC population (A) 1,000 E14.5 iLT-HSCs (purple) or iST-HSCs (blue) were transplanted into lethally irradiated recipients. Peripheral blood erythrocytes (Ery), platelets (Plt), and leukocytes (leuco) were analyzed at 5, 16, and 40 weeks post-transplantation. iLT-HSCs, *n* = 10 recipients, donor cells from 3 pools of litters, 3 experimental days. iST-HSCs, *n* = 11 recipients, donor cells from 3 pools of litters, 3 experimental days. Only comparisons where *p* < 0.05 are displayed. (B) Reconstitution of ABM LSK subsets with 1,000 E14.5 FL iLT-HSCs or iST-HSCs in lethally irradiated recipients after >40 weeks iLT-HSCs, *n* = 4 recipients, donor cells from 3 pools of litters, 3 experimental days. iST-HSCs, *n* = 4 recipients, donor cells from 3 pools of litters, 3 experimental days. Only comparisons where *p* < 0.05 are displayed. (C) Titration of E14.5 iLT-HSCs (i) or iST-HSCs (ii) injected into lethally irradiated recipients. Plts, Ery, and leuco were analyzed after 16 weeks iLT-HSCs, *n* = 4–5 recipient mice per dose, donor cells from 3 pools of litters, 3 experimental days. iST-HSCs, *n* = 6–7 recipients per dose, donor cells from 4 pools of litters, 4 experimental days. (D) Extreme limiting dilution analysis of LT-RU frequency (using data shown in panel D). Dotted lines represent 95% confidence interval for each population. (E) Estimation of LT-RUs in E14.5 iLT-HSC and iST-HSC populations. *n* = 6 embryos from 3 litters. (F) Secondary transplantations using 1/10^th^ (i) or 1/100^th^ (ii) doses of primary recipient bone marrow that had received a 100 cell dose of iLT-HSCs or iST-HSCs. Ery, Plts, and leuco analyzed after 16 weeks. For (i), iLT-HSC, *n* = 6 secondary recipients from 3 primary recipients; iST-HSC, *n* = 9 secondary recipients from 3 primary recipients. For (ii), iLT-HSC, *n* = 6 secondary recipients from 3 primary recipients; iST-HSC, *n* = 8 secondary recipients from 3 primary recipients. All *p* values shown. For (A)–(F), donor-derived reconstitution (Reconstitution %) was determined using donor-specific GFP expression. Dashed line, 1% reconstitution threshold. *p* values calculated from contingency analysis (Fisher’s exact test) of reconstitution outcomes.

We first compared the performance of E14.5 iLT- and iST-HSCs following transplantation into lethally irradiated recipients (the gold-standard LT-RU assay). Analysis of erythroid, platelet, and leukocyte reconstitution at 5, 16, and 40 weeks after transplantation indicated that *bona fide* LT-RUs were present in both populations (Figures 5A and S3). Interestingly, LT-RUs from both iLT- and iST-HSCs reconstituted the entire LSK compartment, including iLT-HSCs (Figure 5B).

To determine LT-RU frequency in the E14.5 iLT- and iST-HSC populations, we transplanted 10, 50, 100, or 1,000 purified cells into lethally irradiated recipients; mice were analyzed after 16 weeks (Figure 5C). Using the extreme limiting dilution method (Hu and Smyth, 2009), we found that LT-RUs were present at a frequency of 1/19 iLT-HSCs (which is in keeping with a previous estimate [Kim et al., 2006]) and 1/34 iST-HSCs (Figure 5D). Considering the absolute number of iLT- and iST-HSCs per E14.5 FL (Figure 1D), we estimated that the iST-HSCs population contains twice as many LT-RUs than the iLT-HSC population (Figure 5E).

To investigate the self-renewal capacity of LT-RUs in the iST-HSC population (herein referred to as iST:LT-RU), 1/10 or 1/100 doses of ABM from primary recipients (that had received 100 donor cells) were transplanted into lethally irradiated secondary recipients. No significant difference in reconstitution was observed between iLT:LT-RU and iST:LT-RU (Figure 5F). Thus, the E14.5 iST-HSC population contains large number of *bona fide* LT-RUs that do not exhibit lineage biases.

### Refinement of the E14.5 FL ST-HSC LT-RU immunophenotype

**Figure 6:**
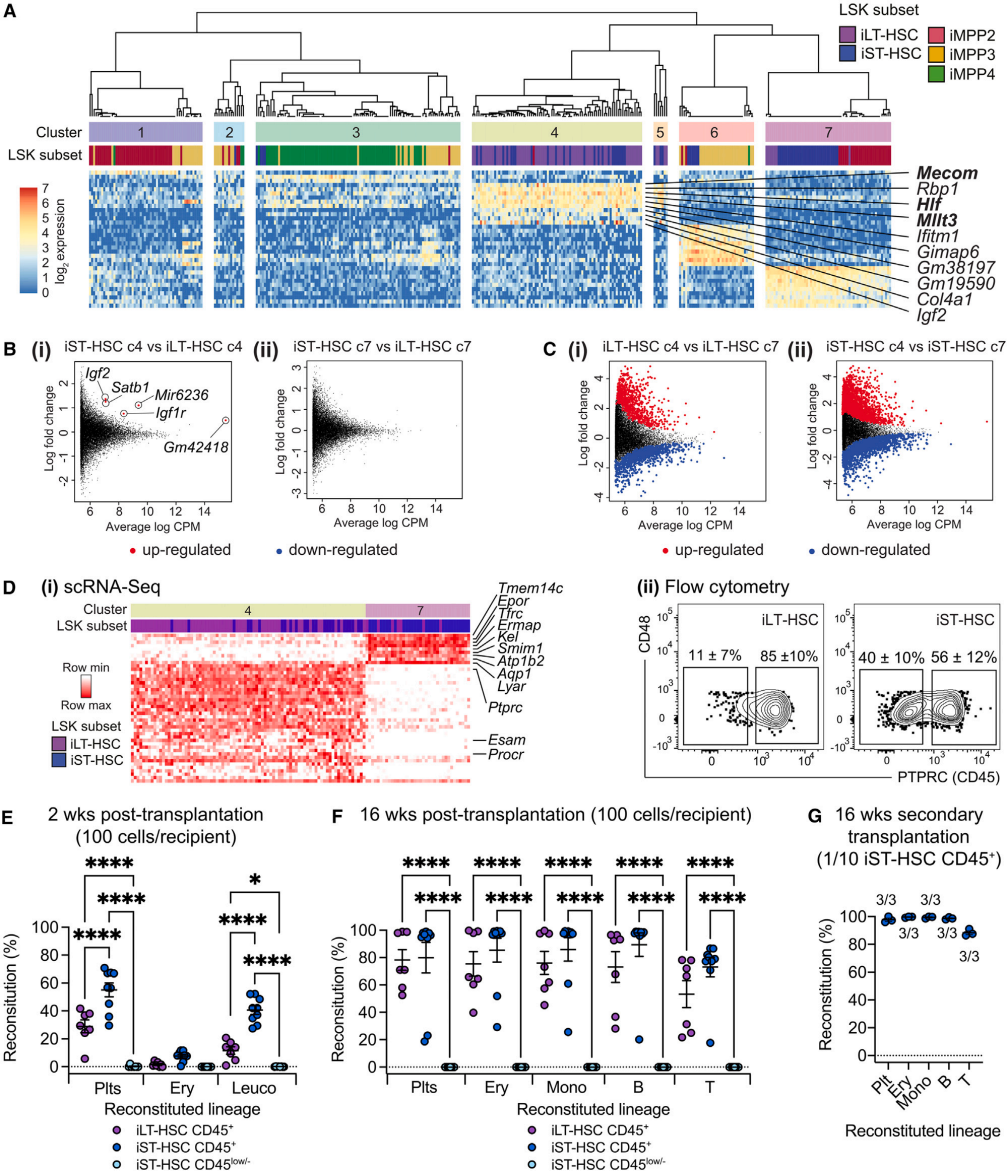
Refinement of the E14.5 iST:LT-RU immunophenotype (A) Heatmap of marker genes for each E14.5 FL LSK transcriptional cluster. (B) Intra-cluster mean average (MA) plots of iLT-HSCs vs. iST-HSCs in cluster 4 (i) and cluster 7 (ii). (C) MA plots of iLT-HSCs in cluster 4 vs. cluster 7 (i) and iST-HSCs in cluster 4 vs. cluster 7 (ii). (D) (i) Heatmap of differentially expressed genes between iST-HSCs in cluster 4 vs. cluster 7. (ii) Representative flow cytometry plots of PTPRC (CD45) expression by iLT-HSCs and iST-HSCs. *n* = 14 embryos from 3 litters, 3 experimental days. Values, mean ± SEM. (E and F) Reconstitution from 100 iLT-HSC CD45^+^, 100 iST-HSC CD45^+^, or 100 iST-HSC CD45^low/-^ cells transplanted into lethally irradiated recipients. Plts, Ery, and Leuco lineages were analyzed after 2 (E) or 16 (F) weeks. ns, not statistically significant. ^∗^, *p* < 0.05. ^∗∗∗∗^, *p* < 0.00005. iLT-HSC CD45^+^, *n* = 7 recipients, donor cells pooled from 2 litters, 2 experimental days. iST-HSC CD45^+^, *n* = 9 recipients, donor cells pooled from 3 litters, 3 experimental days. iST-HSC CD45^low/-^, *n* = 8 recipients, donor cells pooled from 3 litters, 3 experimental days. (G) Secondary transplantations using 1/10^th^ doses of primary recipient bone marrow that had received a 100 cell dose of iST-HSC CD45^+^. Peripheral blood Plt, Ery, and Leuco lineages were analyzed after 16 weeks *n* = 3 secondary recipients from 2 primary recipients. Values, frequency of secondary reconstitution. For (E)–(G), donor-derived reconstitution (Reconstitution %) was determined using donor-specific GFP expression.

We next investigated our E14.5 FL LSK scRNA-seq dataset to understand if a unifying LT-RU transcriptional signature could be identified. Based on similarity matrix analysis, 7 transcriptional clusters were identified; most iLT-HSCs and iST-HSCs segregated into clusters 4 and 7 (Figure 6A). Derivation of signature genes for these clusters indicated that cluster 4 likely contained LT-RUs: *Mecom*, *Hlf*, and *Mllt3* are strongly associated with LT-RU function (Calvanese et al., 2019; Kataoka et al., 2011; Komorowska et al., 2017; Wahlestedt et al., 2017) and were enriched in cluster 4 (Figures 6A and S4; Tables S5 and S6). In contrast, erythroid-associated genes (e.g., *Epor*, *Klf1*, and *Redrum*) were expressed at higher level in cluster 7 (Tables S5 and S6).

Intra-cluster differential gene expression analysis between iLT- and iST-HSCs revealed that cells within clusters were very similar (Figure 6B; Tables S7 and S8), while many genes were differentially expressed between clusters (Figure 6C; Tables S5 and S6). Five genes were found to be significantly differentially expressed between iLT- and iST-HSCs within cluster 4: *Gm42418*, *Igf2*, *Igf1r*, *Mir6236*, and *Satb1* were more highly expressed in iST-HSCs (Figure 6Bi; Table S7). Interestingly, previous studies have shown the following: *in vitro* treatment of FL and ABM iLT-HSCs with IGF2 results in LT-RU expansion (Zhang and Lodish, 2004), level of IGF2 receptor expression correlates with greater hematopoietic reconstitution (Zhang and Lodish, 2004), enforced expression of *Igf2* in ABM iLT-HSCs enhances hematopoietic reconstitution (Thomas et al., 2016), and high levels of *Satb1* expression correlate with improved reconstitution (Doi et al., 2018). This suggests that some degree of functional difference between iLT:LT-RUs and iST:LT-RUs might exist.

Given that LT-RU frequency in the iST-HSC population was half that observed in the iLT-HSC population, to directly compare iST:LT-RUs with iLT:LT-RU, we first aimed to improve iST:LT-RU enrichment. To this end, differential gene expression analysis was performed between iST-HSCs in cluster 4 and cluster 7. Among the significantly upregulated genes in cluster 4, *Ptprc* (which encodes CD45) was found to be strongly upregulated (Figure 6Di; Table S9). Other genes included *Procr* (which encodes EPCR) and *Esam* (Figure 6Di; Table S9), both of which are known to be expressed by E14.5 iLT-HSCs and enrich for LT-RU activity (Balazs et al., 2006; Kent et al., 2009; Yokota et al., 2009). CD45 is expressed by LT-RUs once they have emerged in the E11.5 AGM region (Taoudi et al., 2005, 2008), in the E12.5 yolk sac (Taoudi et al., 2005), and in the E13.5–E15.5 FL (Kent et al., 2009; Taoudi et al., 2005). Thus, we considered CD45 expression to be a promising marker for improved FL iST:LT-RU enrichment. Using flow cytometry we found that 85% ± 10% of iLT-HSCs and 56% ± 12% of iST-HSCs were CD45^+^ (Figure 6Dii).

We next compared the ability of CD45+ iLT-HSCs, CD45+ iST-HSCs, and CD45low/- iST-HSCs to provide hematopoietic reconstitution. To this end, 100 cells of each immunophenotype were transplanted into lethally irradiated recipients. After 2 weeks, CD45 low/- iST-HSCs cells failed to contribute to reconstitution but CD45+ iST-HSCs cells provided robust reconstitution that was significantly greater than that of CD45+ iLT-HSCs (Figure 6E); this was in keeping with the enhanced reconstitution observed with higher IGF2 signaling and *Satb1* expression (Doi et al., 2018; Thomas et al., 2016; Zhang and Lodish, 2004). By 16 weeks, CD45 low/- iST-HSCs did not provide any reconstitution, but both CD45+ iLT-HSCs and CD45+ iST-HSCs contributed equivalently (Figure 6F). Secondary transplantation demonstrated the long-term self-renewal capacity of CD45+ iST-HSC LT-RUs (Figure 6G). As all LT-RUs were contained within the 56% of iST-HSCs that expressed CD45 (Figure 6Dii), we estimated the LT-RU frequency in CD45+ iST-HSCs as ∼ 1/19 cells. This is same as the LT-RU frequency in the iLT-HSC population (Figure 5D).

### iST:LT-RUs have a Flt3-expressing ancestry but are not drHSCs

Developmentally restricted HSCs (drHSCs) (Beaudin et al., 2016) are a component of the E14.5 FL LSK. Similar to LT-HSCs, drHSCs express CD150 and can self-renew in secondary transplantation assays. However, under physiological conditions, drHSCs exhaust between the neonatal period and adulthood. Two features distinguish drHSCs from LT-HSCs, which are as follows.

• From 8 weeks post-transplantation, drHSC-derived reconstitution is lymphoid biased (Beaudin et al., 2016). This bias is transferred upon secondary transplantation (Beaudin et al., 2016).
• drHSCs emerge via an *Flt3*-expressing ancestry. Use of an *Flt3*-cre expressing mouse line elegantly distinguishes LT-HSCs from drHSCs in the E14.5 FL LSK CD150+ compartment (Beaudin et al., 2016).

Our data show that iST:LT-RUs are CD150− and provide lineage-balanced reconstitution and thus are unlikely to be related to drHSCs.

**Figure 7:**
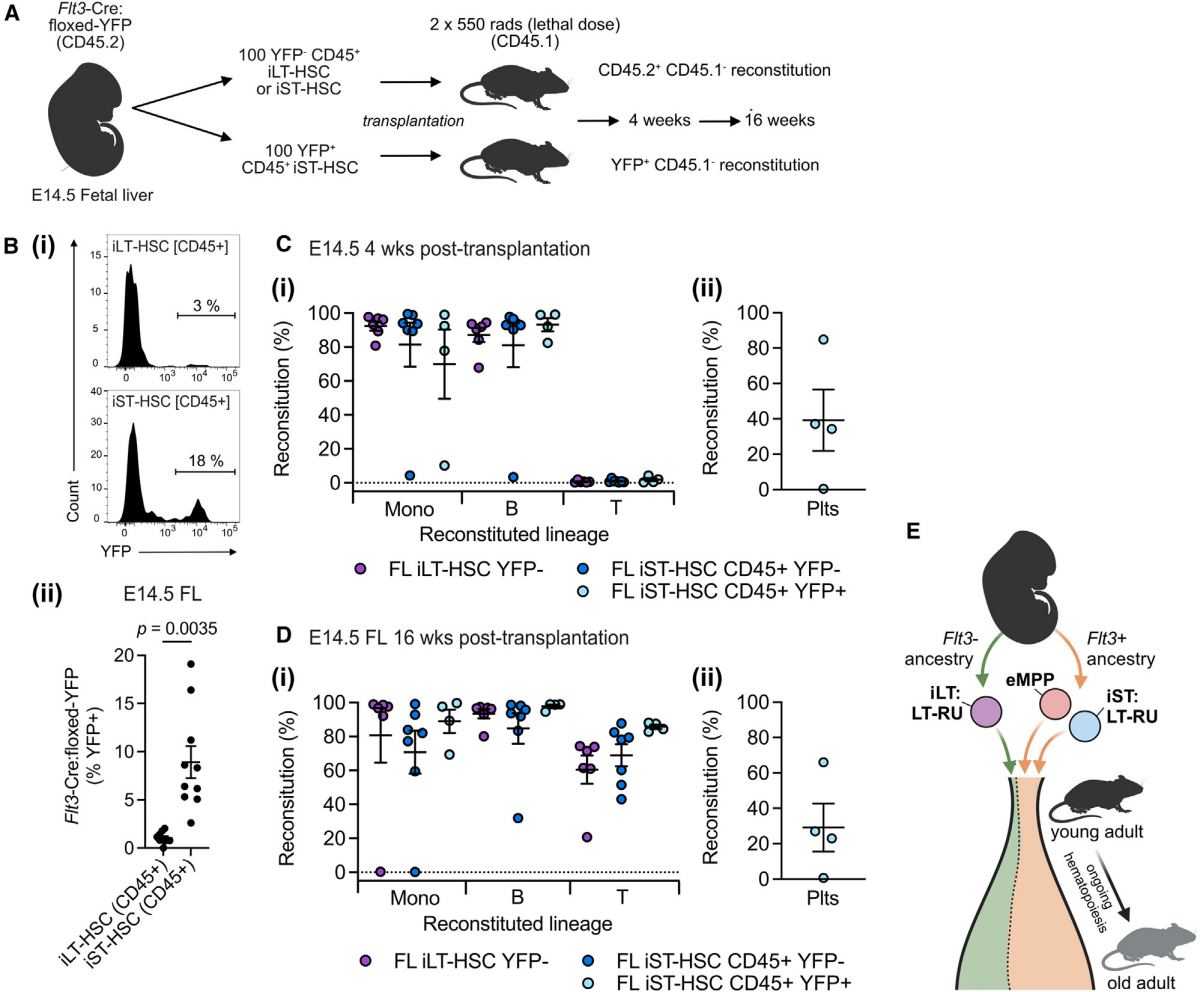
*Flt3*-expressing ancestry of LT-RUs in the iST-HSC population (A) Overview of experimental design. (B–E) (B) (i) Gating strategy used to investigate YFP expression in CD45-expressing iLT-HSCs and iST-HSCs in E14.5 *Flt3*-cre:flox-YFP FLs. (ii) Quantification of YFP expression (% recombination). *n* = 10 embryos, 3 litter, 3 experimental days. Exact *p* values shown. Reconstitution after 4 (C) and 16 (D) weeks. Sufficient numbers of iLT-HSC YFP+ cells could not be purified for transplantation. (i) Contribution to peripheral blood monocytes (Mono), B cells (B), and T cells (T). (ii) Reconstitution of peripheral blood platelet (Plts). Only comparisons where *p* < 0.05 are displayed. iLT-HSC CD45^+^ YFP−, *n* = 6 recipients, donor cells from pooled FL from 3 litters, 3 experimental days. iST-HSC CD45^+^ YFP−, *n* = 7 recipients, donor cells from pooled FL from 5 litters, 5 experimental days. iST-HSC CD45^+^ YFP+, *n* = 4 recipients, donor cells from pooled FL from 4 litters, 4 experimental days. (E) Model of co-contribution of eMPPs (Patel et al., 2022) and iST-HSC[LT-RU]s to ongoing adult hematopoiesis. For C and D, Donor-derived reconstitution (Reconstitution %) determined using CD45.2 expression and YFP expression when possible.

A recent study showed, that under physiological condition, embryonic cells that derive via a *Flt3*-expressing ancestry (and are not iLT-HSCs) contribute to adult hematopoiesis (Patel et al., 2022). These cells were termed long-lived embryonic MPPs. To investigate if iST:LT-RUs were a component of this long-lived embryo-derived axis of native adult hematopoiesis, we investigated E14.5 iLT- and iST-HSCs in *Flt3*-cre mice crossed with floxed-STOP *Rosa26*-EYFP mice (Figure 7A). In keeping with the findings of Patel et al. (2022), we found that CD45+ iST-HSCs were significantly more effectively labeled than iLT-HSCs (Figure 7B). To investigate LT-RUs, 100 YFP+ or YFP− CD45+ iST-HSCs were transplanted into lethally irradiated mice. After 4 weeks, YFP+ and YFP− cells contributed to monocyte and B cell reconstitution (Figure 7C); after 16 weeks, robust monocyte, B cell, and T cell reconstitution was observed (Figure 7D). Thus, iST:LT-RUs of *Flt3*-expressing ancestry were capable of long-term hematopoietic reconstitution without evidence of lymphoid bias.

## Discussion

We have found that E14.5 iMPP subsets are transcriptionally diverse along axes that were predictable based on insight from their adult counterparts (Pietras et al., 2015) and that E14.5 and ABM iMPPs are generally functionally similar. We also found that E14.5 iLT- and iST-HSCs are strikingly distinct from the iMPPs. This was most evident from lentiviral barcoding experiments where complete multilineage fates were a feature of the HSC subsets. It is noted that greater multilineage outcomes from iMPP clones might be detected given more time *in vivo*.

In the adult, iST-HSCs provide poor long-term reconstitution and generally do not reconstitute secondary recipients (thus do not self-renew) (Morita et al., 2010; Oguro et al., 2013; Pietras et al., 2015). We made the surprising discovery that E14.5 iLT- and iST-HSCs were functionally very similar. E14.5 LT-RUs were not only distributed between the iLT- and iST-HSC populations, but most LT-RUs are in the iST-HSC population. Taken together with previous studies, our findings indicate that three LT-RU populations must co-exist in the E14.5 FL: conventional CD150+ iLT:LT-RUs (Kim et al., 2006), CD150+ drHSCs (Beaudin et al., 2016), and CD150− iST:LT-RUs.

Other studies have previously shown that LT-RUs were present among E14.5 CD150− cells (Kent et al., 2009; Kim et al., 2006; Papathanasiou et al., 2009). Because the distribution of LT-RU biomass between CD150+ and CD150− fractions was not quantified, the significance of CD150− LT-RUs was not appreciated. We found that 70% of E14.5 LT-RUs are in the iST-HSC population. Based on our findings, the use of LT-RU-associated markers such as EPCR, ESAM, or CD45 to co-label E14.5 LSK_FLT3- CD48− cells would provide a single unifying LT-RU-enriching immunophenotype. However, this approach would censor potential differences in the developmental pathways of iLT-HSCs and iST-HSCs.

When LT-RUs emerge in the E11.5 AGM region, they do not express CD150 (McKinney-Freeman et al., 2009), and LT-RUs do not express CD150 in the E12.5 placenta (McKinney-Freeman et al., 2009). It is unclear if during continued development *in utero* E14.5 FL iST:LT-RUs would initiate CD150 expression. Although it is presumed that after emergence all LT-RUs will upregulate CD150 expression, this idea is untested. Of note, a previous study suggested that FL iLT-HSCs and iST-HSCs are ontologically distinct lineages that largely maintain segregation and persist into adulthood (Patel et al., 2022). The authors noted that *Flt3*-creERT2-labeled cells from the fetus were significant contributors to hematopoiesis in the adult; it was concluded that this was driven by embryonic MPPs (Patel et al., 2022). Given our findings, it is possible that LT-RUs present in the iST-HSC population could persist into adulthood and contribute to multilineage hematopoiesis (Figure 7E).

## Experimental procedures

### Resource availability

#### Lead contact

Further information and requests should be directed to and will be fulfilled by the lead contact, Samir Taoudi (samir.taoudi@bristol.ac.uk).

#### Materials availability

New reagents were not generated in this study.

#### Data and code availability

Sequencing data have been made available through the GEO database under accession GSE202360. Code will be provided on request.

### Methods

#### Mice

Ly5.1, Ly5.1 x C57BL/6, *Rosa26_*floxed-EYFP, UBC-GFP, and *Flt3*-cre (Benz et al., 2012) mice were maintained as C57BL/6. Noon of the day of a positive vaginal plug check was considered embryonic day (E) 0.5. Adult mice were analyzed between 8 and 16 weeks of age. Experiments were approved by the WEHI animal ethics committee.

#### Fetal tissue collection

The uteri were washed after removal in Ca^2+^/Mg^2+^-free DPBS, 7% fetal calf serum, and 100 units/mL penicillin-streptomycin (fluorescence-activated cell sorting [FACS] wash). Embryos were staged according to Theiler’s criteria. FLs were mechanically dissociated and filtered through a 70 μm sieve.

#### Adult tissue collection

Femurs and tibias were dissected, washed, and crushed in FACS wash with 0.25 mM EDTA. Bone marrow was mechanically dissociated and filtered through a 70 μm sieve. Single-cell suspensions of spleens and thymi were generated by passing organs through a 70 μm sieve. Blood samples were collected in EDTA-containing Microvette tubes.

#### Flow cytometry

KIT-expressing FL or ABM cells were enriched using magnetic separation (Miltenyi Biotech). KIT-enriched cells were stained for flow analysis or sorting. All antibody stains were performed in FACS wash on ice in the dark. Analysis and cell sorting were carried out on a BD LSRFortessa or a BD FACSAria (using an 85 μm nozzle). Viability was accessed with 7AAD. Data were analyzed using FlowJo. See Table S10 for post-sort purity checks. Further details (including antibody information) can be found in the supplemental experimental procedures.

#### Hematopoietic reconstitution assays

Recipient mice were CD45.1 8–10 weeks old. Lethal irradiation was delivered as a 1,100 rads split dose (2 × 550 rads, 3 h gap), and sub-lethal as a single 600 rads dose. Lethally irradiated recipients received 2 × 10^5^ ABM “filler” cells (CD45.1 or CD45.1/2). Donor cells were all CD45.2. Cells were prepared in FACS wash and transplanted via intravenous injection 3 h after irradiation. Recipients were maintained on neomycin for 14–21 days.

#### Single-cell RNA-seq

E14.5 FLs from 9 embryos were pooled from two litters and index-sorted into a 384-well plate. Libraries were prepared using CEL-Seq2 (Amann-Zalcenstein et al., 2020; Hashimshony et al., 2016). Differential expression analyses both between cell types and between cell clusters were carried out using edgeR v3.26.6. For each analysis, biological variation between samples was estimated using edgeR’s estimateDisp function. GO and Kyoto Encyclopedia of Genes and Genomes pathway analyses were performed using limma v3.40.6. Dimension reduction using principal-components analysis, uniform manifold approximation and projection, and clustering was performed using Seurat v3.0.2. Further details are in the supplemental experimental procedures.

#### Lentiviral barcoding

The mCHERRY-expressing SPLINTR library of DNA barcodes used contained ∼70,000 unique barcodes (Fennell et al., 2022). E14.5 LSK subsets were collected from pooled littermates. Barcodes below a threshold of 100 sequencing reads or only detected in one PCR replicate were removed to minimize artifacts. The final number of barcodes present in a given population is a function of biological variation and post-sequencing quality control. Strict filtering of barcode sequencing data is essential to ensure that barcodes analyzed are legitimately clonal. Heatmaps of barcode counts were generated using the R package “heatmap 3.” Finally, the fate of barcodes was determined and correlated with fates computed between different populations using the Pearson correlation coefficient. For lineage biomass (the relative contribution of a barcode to a lineage), barcode frequencies were normalized to sequencing counts per million (CPM). Per cell type (iLT-HSC, iST-HSC, iMPP2, iMPP3, or iMPP4) and outcome (B cell, erythroid cell, monocyte, and neutrophil), we computed CPM sum and then divided this by the total biomass in each cell type. Further details are found in the supplemental experimental procedures.

#### General statistical analysis

Prism 9 (GraphPad) was used for data analysis and graph production. Unless otherwise stated, comparisons between two groups were performed using the Student’s t test (two-way, unpaired) and multiple comparisons with one-way ANOVA using Sidak’s *p* value adjustment for multiple comparisons. “*n*” was used to designate the number of independent experimental mice.

## Supplemental information

Document S1. Figures S1–S4 and supplemental experimental procedures

Data S1. Tables S1–S10

Document S2. Article plus supplemental information
