# Supplementary material for: Clonal analysis of fetal hematopoietic stem/progenitor cells reveals how post-transplantation capabilities are distributed
Source: Stem Cell Reports. 2024 Aug 1;19(8):1189–204. doi: 10.1016/j.stemcr.2024.07.003 (PMC11368694; doi:10.1016/j.stemcr.2024.07.003)
Supplement: Document S1. Figures S1–S4 and supplemental experimental procedures [file mmc1.pdf]

Stem Cell Reports, Volume *19*

Supplemental Information

Clonal analysis of fetal hematopoietic stem/progenitor cells reveals how post-transplantation capabilities are distributed

Olivia J. Stonehouse, Christine Biben, Tom S. Weber, Alexandra Garnham, Katie A. Fennell, Alison Farley, Antoine F. Terreaux, Warren S. Alexander, Mark A. Dawson, Shalin H. Naik, and Samir Taoudi

Supplementary Figures and Legends

Figure S1

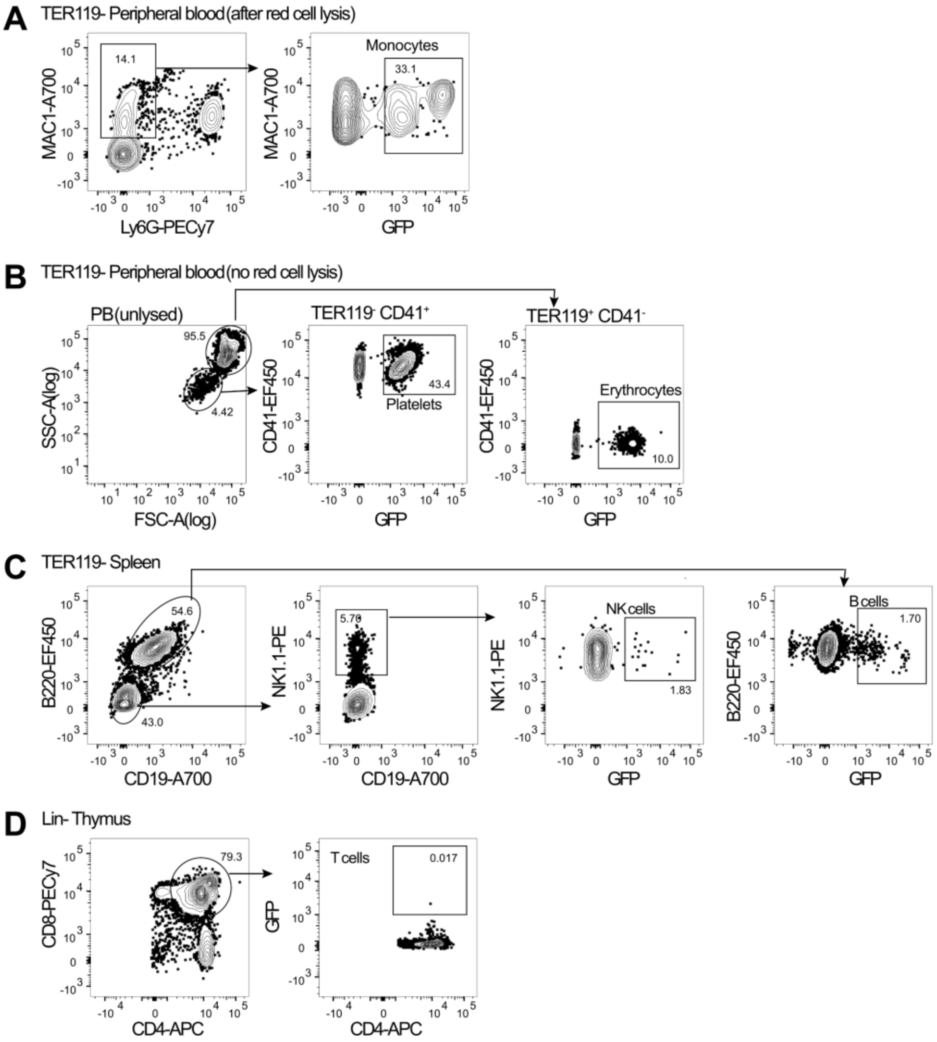

Figure S1. Representative examples for the gating strategy for the analysis of GFP donor-derived hematopoietic reconstitution in sub-lethally irradiated GFP- recipient mice two weeks after transplantation.

Data shown in this example is from the analysis of reconstitution derived from E14.5 fetal liver iST-HSCs. **(A)** Investigation of GFP+ reconstitution of monocytes in recipient (GFP-) peripheral blood. **(B)** Investigation of GFP+ reconstitution of platelets and erythrocytes in recipient (GFP-) peripheral blood. **(C)** Investigation of GFP+ reconstitution of natural killer (NK) and B cells in recipient (GFP-) spleen. **(D)** Investigation of GFP+ reconstitution of T cells in recipient (GFP-) thymus.

Figure S2

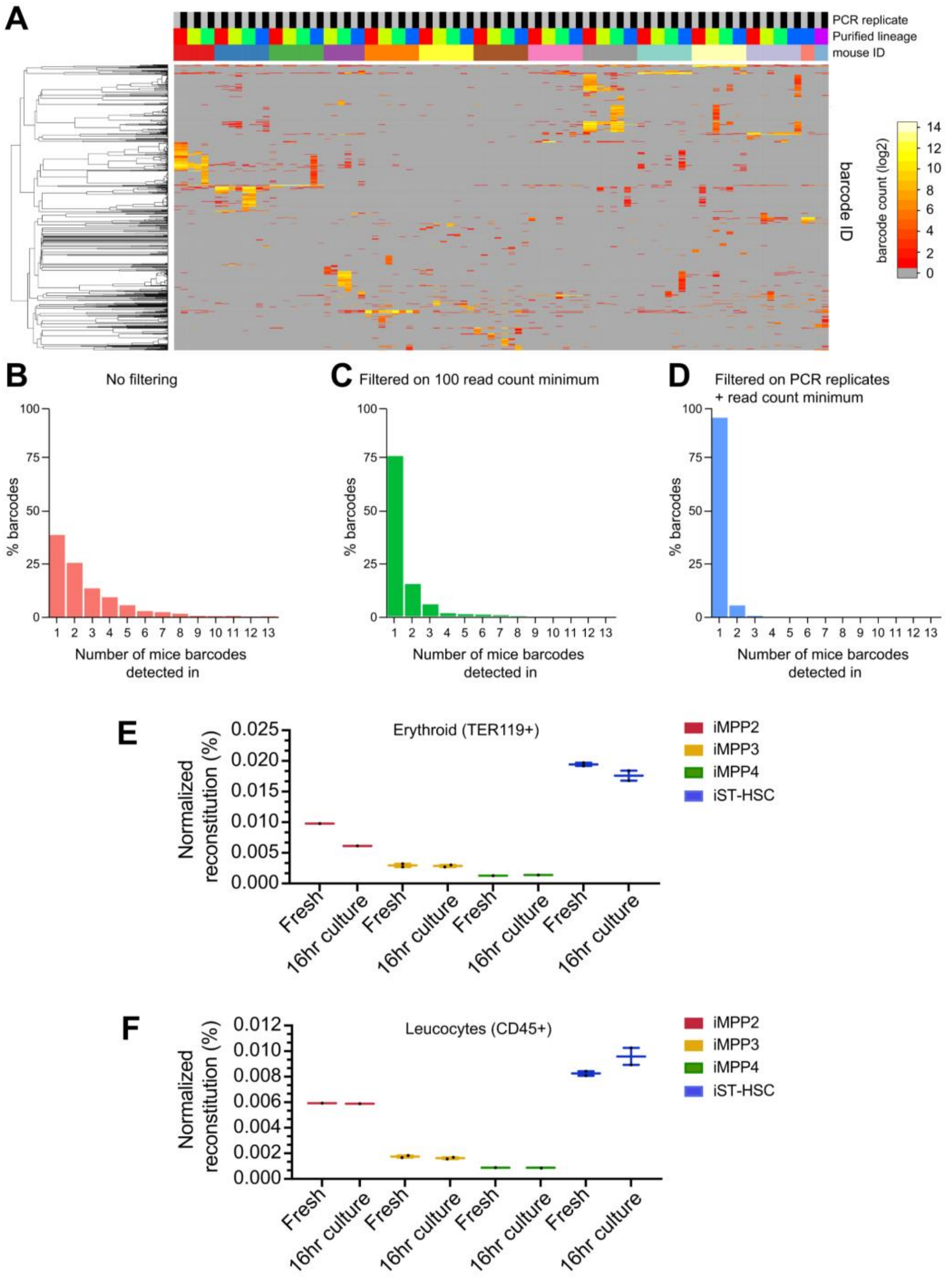
A
PCR replicate
Purified lineage
mouse ID
barcode ID
barcode count (log2)
B
No filtering
% barcodes
Number of mice barcodes detected in
C
Filtered on 100 read count minimum
D
Filtered on PCR replicates + read count minimum
E
Erythroid (TER119+)
Normalized reconstitution (%)
Fresh
16hr culture
iMPP2
iMPP3
iMPP4
iST-HSC
F
Leucocytes (CD45+)

Figure S2. Quality control for lentiviral barcoding experiments.

(A) Heatmap of all barcode sequences detected before filtering in the experimental cohort of 14 animals. Mouse ID are color-coded. Purified lineage: Red: B cells, Yellow: Erythrocytes, Green: Monocytes, Blue: Neutrophils, Purple: blank. 2 PCR replicates (grey/black) were performed for each sample.

(B – D) Barcode sharing between all cohorts of experimental mice with no data filtering **(B)**, after removal of low read counts ("min_read") **(C)**, and after PCR replicate filtering ("PCR rep") and "min_read" filtering **(D)**. Data analyzed in this study were based on "PCR rep" + "min_read" filtering. This shows that >95% bona fide barcodes are detected in one mouse only, suggesting that repeated usage of barcodes is unlikely.

(E – F) Normalized reconstitution per cell two weeks after transplantation of freshly isolated E14.5 FL LSK subsets or E14.5 FL LSK subsets following 16 hrs of *in vitro* culture. **(E)** Reconstitution in the erythroid lineage. **(F)** Reconstitution in leucocytes. iMPP2 n = 1 recipient. iMPP3, n = 2 recipients. iMPP4, n = 1 recipient. iST-HSC, n = 2 recipients.

Figure S3

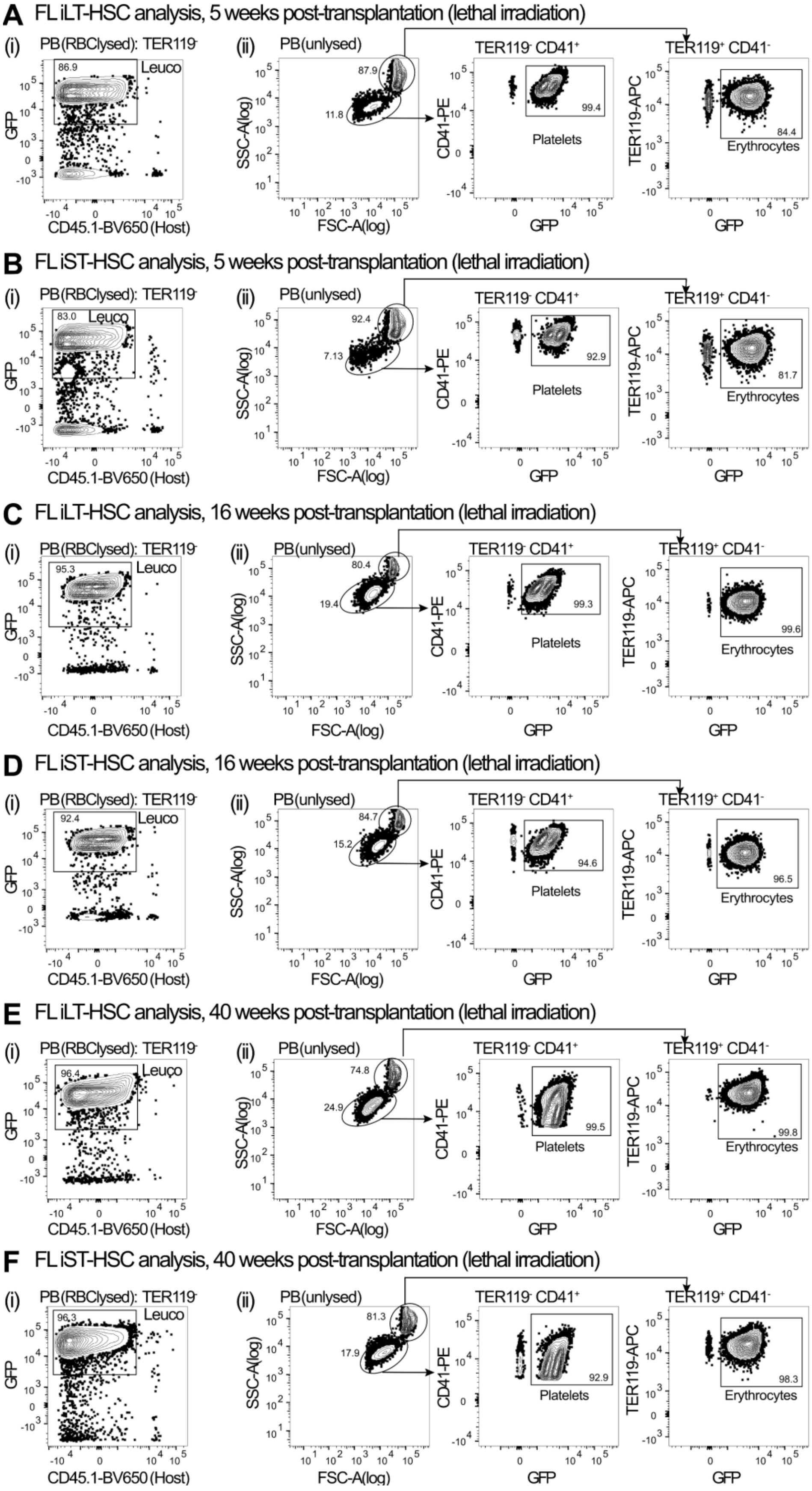
A FL iLT-HSC analysis, 5 weeks post-transplantation (lethal irradiation)
B FL iST-HSC analysis, 5 weeks post-transplantation (lethal irradiation)
C FL iLT-HSC analysis, 16 weeks post-transplantation (lethal irradiation)
D FL iST-HSC analysis, 16 weeks post-transplantation (lethal irradiation)
E FL iLT-HSC analysis, 40 weeks post-transplantation (lethal irradiation)
F FL iST-HSC analysis, 40 weeks post-transplantation (lethal irradiation)
(i) PB(RBClysed): TER119-
(ii) PB(unlysed)
TER119- CD41+
TER119+ CD41-
Leuco
Platelets
Erythrocytes
GFP
CD45.1-BV650 (Host)
SSC-A(log)
FSC-A(log)
CD41-PE
TER119-APC

Figure S3. Representative examples of the gating strategy used for the identification of GFP+ donor-derived reconstitution in lethally irradiated GFP- recipient mice 5, 16, and 40 weeks after transplantation.

(A) At 5 weeks post-transplantation, identification of GFP+ leucocytes (i), platelets (ii), and erythrocytes (ii) in recipient peripheral blood from E14.5 iLT-HSC.

(B) At 5 weeks post-transplantation, identification of GFP+ leucocytes (i), platelets (ii), and erythrocytes (ii) in recipient peripheral blood from E14.5 iST-HSC.

(C) At 16 weeks post-transplantation, identification of GFP+ leucocytes (i), platelets (ii), and erythrocytes (ii) in recipient peripheral blood from E14.5 iLT-HSC.

(D) At 16 weeks post-transplantation, identification of GFP+ leucocytes (i), platelets (ii), and erythrocytes (ii) in recipient peripheral blood from E14.5 iST-HSC.

(E) At 40 weeks post-transplantation, identification of GFP+ leucocytes (i), platelets (ii), and erythrocytes (ii) in recipient peripheral blood from E14.5 iLT-HSC.

(F) At 40 weeks post-transplantation, identification of GFP+ leucocytes (i), platelets (ii), and erythrocytes (ii) in recipient peripheral blood from E14.5 iST-HSC.

Figure S4

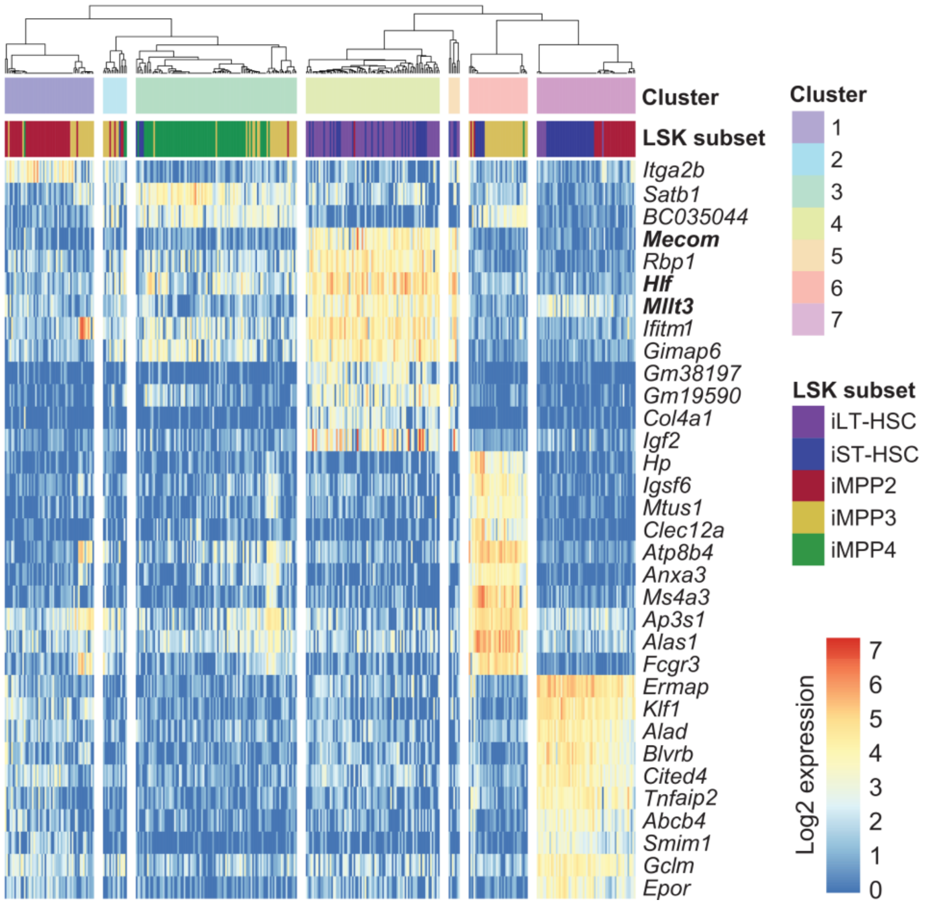

Figure S4. Signature genes of E14.5 FL LSK transcriptional clusters. Heatmap of E14.5 FL LSK transcriptional cluster signature genes (linked to Figure 6A).

Supplementary Tables

Table S1. Differential gene expression between E14.5 FL iMPP4 and iMPP3

Table S2. Differential gene expression between E14.5 FL iMPP4 and iMPP2

Table S3. Differential gene expression between E14.5 FL iMPP3 and iMPP2

Table S4. Genes associated with iMPP-derived GO terms

Table S5. Inter-cluster 4 and 7 differential gene expression between E14.5 FL iLT-HSCs

Table S6. Inter-cluster 4 and 7 differential gene expression between E14.5 FL iST-HSCs

Table S7. Intra-cluster 4 differential gene expression between E14.5 FL iLT-HSCs and iST-HSCs

Table S8. Intra-cluster 7 differential gene expression between E14.5 FL iLT-HSCs and iST-HSCs

Table S9. Inter-cluster 4 and 7 differential gene expression - genes with plasma membrane associated products

Table S10. Summary of post-sort purity checks

Supplementary Experimental Procedures

Flow cytometry

In the E14.5 fetal liver and adult bone marrow, LT-HSCs were defined as Lin-KIT+SCA1+FLT3-CD48-CD150+; ST-HSCs Lin-KIT+SCA1+FLT3-CD48-CD150-; MPP4s Lin-KIT+SCA1+FLT3+CD48+CD150-; MPP3s Lin-KIT+SCA1+FLT3-CD48+CD150-; MPP2s Lin-KIT+SCA1+FLT3-CD48+CD150+. Lineage cocktail contained; Ter119, B220, MAC1, CD3ε+, CD19, CD4, CD8, Ly6G, NK1.1. Anti-Mac1 antibody was omitted in the lineage cocktail for fetal liver preparations to avoid excluding some of the Lin-KIT+SCA1+ (Morrison et al., 1995). Differentiated hematopoietic lineages were defined as: erythroid cells TER119+; B cells TER119-MAC1- B220+ CD19+; Monocytes TER119- MAC1+ Ly6G- SSC_A low; Neutrophils TER119-MAC1+ Ly6G+; Platelets TER119- CD41+; Natural killer cells TER119- MAC1- B220- CD3ε-NK1.1+; T cells TER119- MAC1- B220- NK1.1- CD3ε+.

Antibody clones used, fluorochromes used, and suppliers are: anti-SCA1, PE-Cy7 (clone D7, BD Biosciences); anti-CKIT, APC (2B8), BioLegend; anti-CD150 BV421 (clone TC15-12512.2, BioLegend); anti-FLT3 PE (clone A2F10, BioLegend); anti-CD48 PE-Cy7 (clone HM48-1, BioLegend); anti-CD45 Biotin (clone 30-F11, BioLegend); anti-TER119 AF700 (clone TER119, WEHI); anti-B220 AF700 (clone RAS6B2, WEHI); anti-CD19 AF700 (clone 1D3, WEHI); anti-CD8 AF700 (clone 53-6.7, WEHI); anti-CD4 AF700 (clone GK1.5, WEHI); anti-CD2 AF700 (clone RM2.1, WEHI); anti-CD3ε AF700 (clone 145-2C11, WEHI); anti-MAC1 PE (clone M1/70, BD Biosciences); anti-MAC1 PE (clone M1/70, BD Biosciences); anti-CD42D APC (clone 1C2, BioLegend); anti-CD41 PE-Cy7 (clone MWReg30, BioLegend); anti-NK1.1 PE (clone PK136, BioLegend). BV711-conjugated Streptavidin (BioLegend) was used to detect Biotin tags. 7AAD was purchased from Life Technologies.

Single cell RNA-Seq

Sorts were undertaken using aseptic protocols and using a 100 μm nozzle. For flow alignment, 1 bead coated with Horse Radish Peroxidase was sorted into 1.2 μL of horse-radish peroxidase substrate before and after sort controls. Colorimetric assessment confirmed proper alignment of

the cell sorter. Libraries were prepared using CELSeq2 (Hashimshony et al., 2016) with additional optimizations, as described in (Amann-Zalcenstein et al., 2020). Single cells were sorted into plates containing a primer/lysis mix (dNTPs, ERCC and SUPERase Inhibitor). Plates were centrifuged for 1 min at 1,200 x *g* and immediately frozen down at –80°C until further processing. Sequencing reads were aligned and mapped to the mm10 mouse genome and ERCC spike-in sequences using Rsubread (Liao et al., 2019). GRCm38.88_chr annotation was then added. The data was demultiplexed and reads overlapping genes were summarized into counts using scPipe v1.6.0 (Tian et al., 2018). All subsequent analyses were performed in R version 3.6.0. Seven cells were identified as outliers based on number of genes detected, total gene counts, ERCC percentage, mitochondrial gene percentage and ribosomal gene percentage, and were excluded leaving 368 cells for downstream analysis. Gender-related genes were removed to avoid potential gender biases in the analysis. Lowly expressed genes were also filtered out such that 15590 genes showing an average count greater than 1 in at least 10 cells were retained. Normalisation factors were computed by deconvolution using scran v1.12.1 (Lun et al., 2016) and subsequently used to calculate log-transformed normalised expression values. Gene-specific variance of the biological and technical components of the data were then estimated. Cell cycle phases were predicted using the cyclone function in scran. Generalized log-linear models were then fitted to the count data, incorporating an adjustment for cell cycle phase. edgeR's likelihood ratio test pipeline was applied to identify differentially expressed genes between the cell populations (McCarthy et al., 2012). The false discovery rate (FDR) was controlled below 5% using the Benjamini Hochberg method. Code used for analysis will be made available on request.

Lentiviral barcoding

A maximum of 3 x 10^4 purified E14.5 FL LSK cells were transduced with SPLINTR virus in 96-well plates via spin-infection (90 mins at 1250 x *g*) at a multiplicity of infection of 0.01 - 0.02 (this provided 1 – 2% mCHERRY+ cells) to limit multiple integrations of the barcode per cell. Cells were washed and then injected intravenously into recipient mice. After two weeks mCHERRY+ barcoded cells were retrieved from the spleens of recipient mice two-weeks post transplantation. Spleens were stained with markers for the isolation of monocytes (TER119$^-$MAC1$^+$Ly6G$^-$ [low side-

scatter]), neutrophils ($TER119^-MAC1^+LY6G^+$), erythroblasts ($TER119^+CD44^+$), and B cells ($TER119^-MAC1^-B220^+$) (Figures S4, S5). $mCHERRY^+$ cell populations were sorted and spun into pellets of no more than 5×10^5 cells. Pellets were resuspended in 40 µL Direct PCR Lysis Reagent (Cell) with 0.5 mg/mL Proteinase K. Cells underwent lysis at 55°C for 2 hrs, followed by 85°C for 30 mins and 95°C for 5 mins. Libraries were generated as outline in (Naik et al., 2013). Raw sequencing FASTQ files were processed using custom C++ code to count SPLINTR barcode reads in each sample, including exclusively barcodes that aligned to a previously generated reference library. All subsequent analyses were performed using the statistical computing language R. Counts below a threshold of 100 reads were set to zero.

Note that the final number of barcodes present in a given population is a function of: (1) Biological variation: this includes the abundance of the population (which dictates the number of cells that could carry a barcode), and the range of burst capabilities of the engrafting clones (the biomass contribution). Because little difference in hematopoietic reconstitution between freshly isolation and short-term cultures (Figure S3), the recovery of a large/small number of barcodes (clones) likely represents a biological feature of the population investigated. (2) Quality control measures: as shown in Figure S2, strict filtering of barcode sequencing data is essential to ensure that barcodes analysed are legitimately clonal. Scripts used for barcode analysis will be provided on request.

Supplementary References

Amann-Zalcenstein, D., Tian, L., Schreuder, J., Tomei, S., Lin, D.S., Fairfax, K.A., Bolden, J.E., McKenzie, M.D., Jarratt, A., Hilton, A., et al. (2020). A new lymphoid-primed progenitor marked by Dach1 downregulation identified with single cell multi-omics. Nat Immunol *21*, 1574-1584. 10.1038/s41590-020-0799-x.

Hashimshony, T., Senderovich, N., Avital, G., Klochendler, A., de Leeuw, Y., Anavy, L., Gennert, D., Li, S., Livak, K.J., Rozenblatt-Rosen, O., et al. (2016). CEL-Seq2: sensitive highly-multiplexed single-cell RNA-Seq. Genome Biol *17*, 77. 10.1186/s13059-016-0938-8.

Liao, Y., Smyth, G.K., and Shi, W. (2019). The R package Rsubread is easier, faster, cheaper and better for alignment and quantification of RNA sequencing reads. Nucleic acids research *47*, e47-e47.

Lun, A.T., McCarthy, D.J., and Marioni, J.C. (2016). A step-by-step workflow for low-level analysis of single-cell RNA-seq data with Bioconductor. F1000Research *5*.

McCarthy, D.J., Chen, Y., and Smyth, G.K. (2012). Differential expression analysis of multifactor RNA-Seq experiments with respect to biological variation. Nucleic Acids Res *40*, 4288-4297. 10.1093/nar/gks042.

Morrison, S.J., Hemmati, H.D., Wandycz, A.M., and Weissman, I.L. (1995). The purification and characterization of fetal liver hematopoietic stem cells. Proc Natl Acad Sci U S A *92*, 10302-10306. 10.1073/pnas.92.22.10302.

Naik, S.H., Perie, L., Swart, E., Gerlach, C., van Rooij, N., de Boer, R.J., and Schumacher, T.N. (2013). Diverse and heritable lineage imprinting of early haematopoietic progenitors. Nature *496*, 229-232. 10.1038/nature12013.

Tian, L., Su, S., Dong, X., Amann-Zalcenstein, D., Biben, C., Seidi, A., Hilton, D.J., Naik, S.H., and Ritchie, M.E. (2018). scPipe: A flexible R/Bioconductor preprocessing pipeline for single-cell RNA-sequencing data. PLoS Comput Biol *14*, e1006361. 10.1371/journal.pcbi.1006361.
